# Molecular profiling of endometrial carcinoma precursor, primary and metastatic lesions suggests different targets for treatment in obese compared to non-obese patients

**DOI:** 10.18632/oncotarget.2675

**Published:** 2014-11-04

**Authors:** Anna Berg, Erling A. Hoivik, Siv Mjøs, Frederik Holst, Henrica M. J. Werner, Ingvild L. Tangen, Amaro Taylor-Weiner, William J. Gibson, Kanthida Kusonmano, Elisabeth Wik, Jone Trovik, Mari K. Halle, Anne M. Øyan, Karl-Henning Kalland, Andrew D. Cherniack, Rameen Beroukhim, Ingunn Stefansson, Gordon B. Mills, Camilla Krakstad, Helga B. Salvesen

**Affiliations:** ^1^ Department of Clinical Science, Center for Cancer Biomarkers, University of Bergen, Norway; ^2^ Department of Gynecology and Obstetrics, Haukeland University Hospital, Norway; ^3^ Computational Biology Unit, University of Bergen, Norway; ^4^ Department of Pathology, Haukeland University Hospital, Norway; ^5^ Department of Clinical Medicine, Center for Cancer Biomarkers, University of Bergen, Norway; ^6^ Department of Microbiology, Haukeland University Hospital, Norway; ^7^ The Broad Institute of Harvard and MIT, Cambridge, Massachusetts, United States of America; ^8^ Department of Cancer Biology and Department of Medical Oncology, Dana-Farber Cancer Institute, Boston, Massachusetts, United States of America; ^9^ Harvard Medical School, Boston, Massachusetts, United States of America; ^10^ Department of Systems Biology, MD Anderson Cancer Center, Houston Texas

**Keywords:** endometrial carcinoma, endometrial hyperplasia, metastasis, body mass index, PI3Kinase

## Abstract

Obesity is linked to increased incidence of endometrioid endometrial cancer (EEC) and complex atypical hyperplasia (CAH). We here explore pattern and sequence of molecular alterations characterizing endometrial carcinogenesis in general and related to body mass index (BMI), to improve diagnostic stratification and treatment strategies. We performed molecular characterization of 729 prospectively collected EEC and CAH. Candidate biomarkers were identified in frozen samples by whole-exome and Sanger sequencing, oligonucleotide gene expression and Reverse Phase Protein Arrays (investigation cohort) and further explored in formalin fixed tissues by immunohistochemistry and Fluorescent in Situ Hybridization (validation cohort). We here demonstrate that *PIK3CA* mutations, PTEN loss, PI3K and KRAS activation are early events in endometrial carcinogenesis. Molecular changes related to *KRAS* activation and inflammation are more common in obese CAH patients, suggesting different prevention and systemic treatment strategies in obese and non-obese patients. We also found that oncoprotein Stathmin might improve preoperative diagnostic distinction between premalignant and malignant endometrial lesions.

## INTRODUCTION

Cancer of the corpus uteri is the most frequent gynecological cancer in developed countries, globally affecting more than 140, 000 new women each year [1]. Approximately 80% of these patients present with the endometrioid subtype of endometrial carcinoma (EEC) [2]. Endometrial hyperplasia (EH), and in particular complex atypical hyperplasia (CAH) are considered to be precursor lesions for EEC [3].

Both EEC and CAH are associated with factors known to increase the level of biological available estrogen or an excess of estrogen relative to progesterone [3]. After menopause, adipose tissue becomes the primary source of estrogen, with levels correlating with increased body mass index (BMI) [4]. The obesity epidemic in the industrialized world has been linked to the increase in EEC and its precursor lesions [2, 5].

Premalignant endometrial lesions are commonly classified according to the WHO classification system [6]. This classification system has shown poor reproducibility and high risk of concurrent carcinoma or cancer progression [7-9]. One key clinical challenge in treating endometrial carcinoma precursor lesions is that some women with CAH are premenopausal and in childbearing age [10]. These aspects have stimulated the search for biomarkers to improve the distinction of premalignant from malignant lesions for these patients, and to learn more about the biology behind cancer progression. Recently the WHO classification system was revised and hopefully this will improve the diagnostic accuracy [11]. Still, the knowledge about the molecular pattern is scarce, and no biomarker is yet in widespread clinical use to date.

In particular for women wanting to preserve fertility and those medically inoperable, this is an unmet need. On this background, we aimed to explore CAH for biomarkers with a potential to improve diagnostic accuracy. Identifying targets for therapy, potentially improving systemic treatment strategies, would also be important. We have therefore performed a comprehensive molecular profiling of a unique collection of fresh frozen EEC precursor-, primary- and metastatic lesions in parallel, to explore the pattern and sequence of molecular alterations characterizing the disease progression in general and related to BMI in specific. Candidate markers were further explored in a larger sample collection of formalin fixed paraffin embedded (FFPE) tumor tissue for potential validation. Finally, validated markers were tested for their ability to improve the preoperative detection of malignant disease in patients with concordant (CAH/CAH) versus discordant (CAH/EEC) preoperative and postoperative histologic diagnoses.

## RESULTS

Distribution of clinical characteristics for patients diagnosed by total hysterectomy included in this study is presented in Supplementary Table S1. The investigation cohort consists of 139 EEC and 18 CAH patients with fresh frozen tissue available for the comprehensive molecular profiling and the validation cohort of 494 EEC and 77 CAH patients. Patients with CAH were significantly younger and more often premenopausal compared to EEC patients in the validation cohort. A similar pattern was seen for the investigation cohort, although not statistically significant, probably due to the smaller sample set (Supplementary Table S1).

### Missense *PTEN* and *PIK3CA* mutations are frequent in CAH

**Figure 1:**
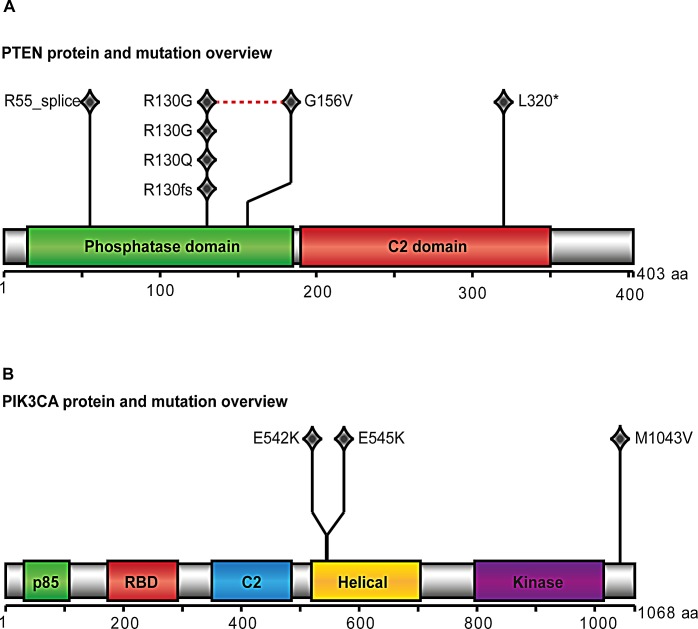
PTEN and PIK3CA protein and mutation view Results from whole exome sequencing of ten cases with complex atypical hyperplasia (CAH): *PTEN* (A) and *PIK3CA* (B) were significantly mutated indicated by protein view in the diagram. One diamond indicates one mutation. The red dotted line in *PTEN* overview indicates two mutations found in same case. Across the two proteins, *PTEN* L320* and PIK3CA E542K is found within same case, as is *PTEN* R55_splice and *PIK3CA* E545K.

*PTEN* and *PIK3CA* were found to be significantly mutated and present in five and three of ten CAHs subjected to whole exome sequencing (WES), respectively (Figure 1). Two cases had coexisting mutations in *PIK3CA* and *PTEN*. All somatic mutations and details regarding the mutations found in the 10 CAH cases assessed by WES are included in Supplementary Data. Other mutations known to occur in premalignant endometrial lesions, like *CTNNB1* and *KRAS* mutations were not detected. The mutation rate in CAH lesions is low compared to primary endometrial cancer lesions [12].

As the majority of *PIK3CA* mutations have previously been reported to occur in exons 9 and 20 [13], these were characterized by Sanger sequencing in additional DNA from fresh frozen tissue available from 18 samples with CAH (of which 8 were included in the WES analyses) and 228 primary EEC lesions. Four (22.2%) CAH cases were found to have missense mutations in exon 9, in addition to one silent mutation in exon 20. Details regarding the mutations in *PIK3CA* are listed for CAH cases in Supplementary Table S2. Two cases were found to have *PIK3CA* mutations by both methods, revealing the same sequence alterations, and located at known *PIK3CA* mutation hotspot sites (E545K and E542K). One overlapping case had *PIK3CA* mutations in exon 20 by WES without the mutation being confirmed by Sanger sequencing, suggesting different detection ranges, with Sanger sequencing requiring a higher mutant allele frequency for detection. The *PIK3CA* mutation frequency in CAH was similar to what was found for grade 1 through 3 in the 228 primary EEC lesions investigated (Supplementary Table S3).

### *PIK3CA* amplifications are infrequent in CAH and increase with dedifferentiation

Fifty-five CAH lesions were further assessed for *PIK3CA* copy number alterations by Fluorescent in Situ Hybridization (FISH). The mean *PIK3CA*/CEP3 Dual Colour Probe ratio was 1.02, ranging from 0.95 to 1.14 in CAH. Gene/centromere ratio was defined as increased if exceeding 1.3 [14] or an average of gene copy number above 2.6 per nucleus for absolute copy number increase. Of the 435 cases with EEC assessed for *PIK3CA* copy number by FISH for comparison, 8.7% demonstrated increased copy number, with the highest proportion of *PIK3CA* amplified cases in EEC grade 3 lesions (Supplementary Table S3). This difference in copy number between CAH and EEC was statistically significant (p=0.01), and in contrast to the similar proportion of *PIK3CA* mutations detected in CAH and different grades of primary EEC lesions (Supplementary Table S3).

### PI3K pathway activation and PTEN loss occur early in endometrial carcinogenesis

**Figure 2:**
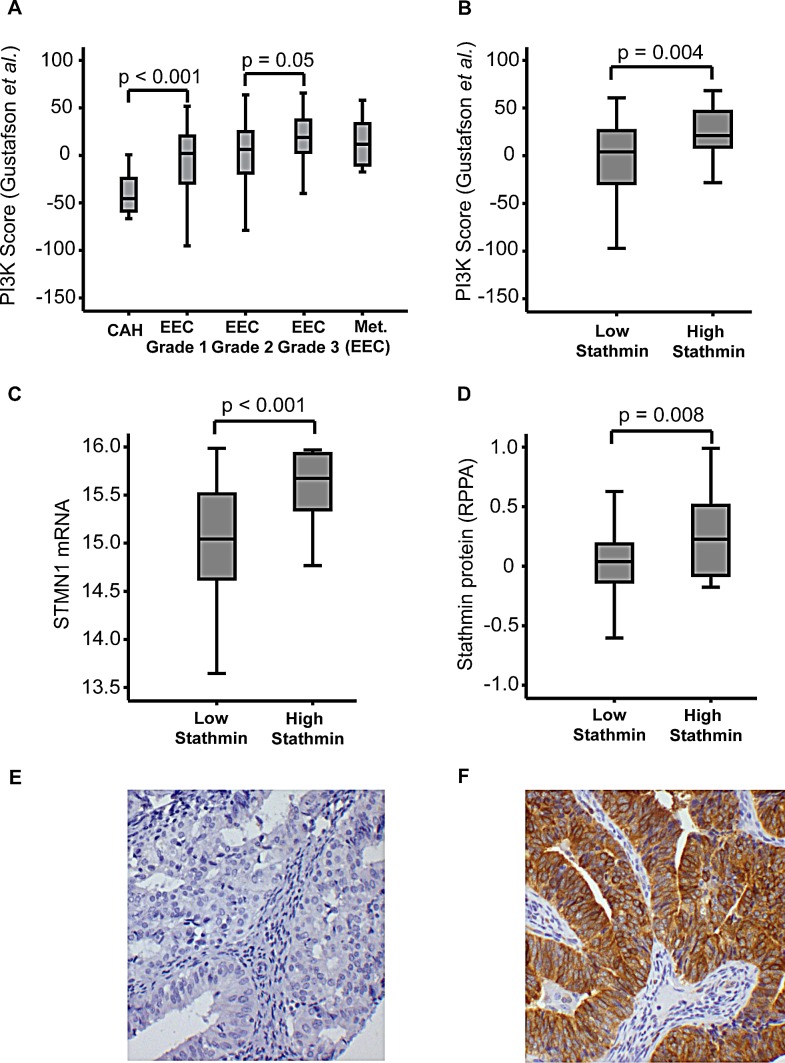
PI3K mRNA signature score and Stathmin protein expression The PI3K mRNA signature score defined by Gustafson *et al*. [18] (A) in complex atypical hyperplasia (CAH), primary endometrioid endometrial carcinomas (EEC), stratified for grade 1, grade 2 and grade 3 and metastatic lesions from EEC primary tumors. Stathmin protein expression assessed by immunohistochemistry (IHC, Cell Signaling #3352) correlated to PI3K mRNA score (B), *STMN1* mRNA level (C) and protein level assessed by Reverse Phase Protein Array (D) in EEC and CAH patients. Examples of Stathmin immunological tissue staining by IHC, showing low (E) and high expression (F). 20x magnification applied.

The PI3K signaling pathway is known to be important in cancer initiation and progression through many mechanisms such as cell growth and cell survival [15]. PI3K activation is influenced by multiple changes in endometrial cancer, including most frequently PTEN loss of function, *PIK3CA* mutations and *PIK3CA* amplification [15-17]. On this background we further explored mRNA expression levels of an established gene signature representing the PI3K pathway [18], to compare the PI3K signaling activity in CAH to EEC lesions grade 1, 2 and 3 and metastatic lesions from EEC primary tumors (Figure 2). We found a highly significant increase in PI3K pathway signaling from CAH to EEC grade 1 (p<0.001). The increased PI3K pathway activation in EEC grade 1 samples could be due to higher glandular purity in these lesions compared to CAH samples. To better control the potential stromal contamination in CAH, we explored the validation cohort by immunohistochemical (IHC) protein staining of the epithelial component for the oncoprotein Stathmin, suggested as a surrogate marker for PI3K and PTEN dysregulation in endometrial and breast cancers [16, 19]. There was a highly significant association between Stathmin protein expression and PI3K activation score from overlapping specimens (p=0.004) (Figure 2). In parallel with evaluating Stathmin protein levels by IHC, Stathmin levels were also assessed by Reverse Phase Protein Arrays (RPPA). Protein levels by both methods were found to be highly significantly correlated with *STMN1* mRNA level (p<0.001 and p=0.008, respectively) (Figure 2), supporting that higher level of stromal contamination in CAH is unlikely to be the sole explanation for the differences in PI3K activation levels demonstrated for CAH and EEC. We also explored immunohistochemical staining for phosphorylated Stathmin (pStathmin(S38)). We found it to be highly correlated with Stathmin protein expression (p<0.001), as reported previously [20]. However this marker was related to the PI3K activation score only with borderline significance (p=0.1).

**Figure 3:**
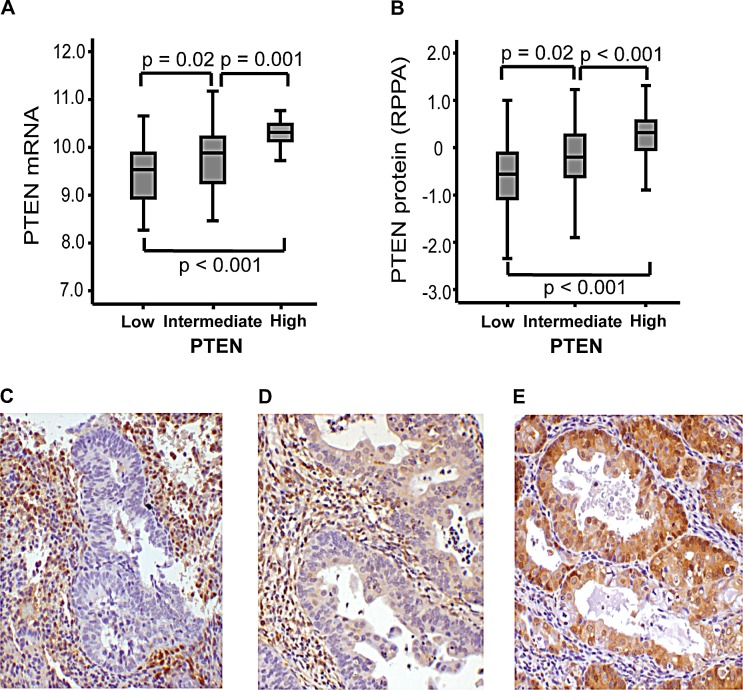
Immunohistochemical staining of PTEN associates with mRNA and protein RPPA-expression PTEN protein level by immunohistochemistry (IHC, Cell Signaling #9188) is significantly associated with *PTEN* mRNA expression in clinical samples from patients with complex atypical hyperplasia and endometrioid endometrial cancer (A) and PTEN protein level assessed by Reverse Phase Protein Array (B). Examples of PTEN protein expression by IHC, showing low (C), intermediate (D) and high (E) staining. 20x magnification applied.

Also, PTEN protein expression was correlated to *PTEN* mRNA level, evaluated both by IHC and RPPA, supporting the feasibility for assessment of PTEN status by these methods for the applied antibody (Figure 3). The correlation between PTEN protein and mRNA expression was further supported in results from Significance Analysis of Microarrays (SAM), presenting *PTEN* as the one top-ranked gene differentially expressed in high versus low PTEN protein expression groups. We found no difference in *PTEN* mRNA expression or expression of PI3K signature level for mutated compared to wild type status for *PIK3CA*.

After validating that Stathmin protein staining reflected PI3K signaling and PTEN protein staining *PTEN* mRNA level in clinical samples, we explored Stathmin as a surrogate marker for PI3K signaling in the independent validation cohort. In line with the observed increase in PI3K signaling from CAH to EEC, we observe a significant increase in PI3K signaling, measured by increased Stathmin protein level from premalignant to malignant lesions in this independent validation cohort (p<0.001) (Table 1). In the same patient cohort we find that PTEN protein loss is more frequent in CAH than EEC (p<0.001) (Table 1).

**Table 1:** Histological type and grade correlated to hormone receptor status, Stathmin and PTEN protein expression in a total of 57 complex atypical hyperplasia (CAH) and 408 endometrioid endometrial carcinoma (EEC) lesions with representative data for type and grade

	Stathmin^a^ n(%)		PTEN^b^ n (%)			ERα^c^ n (%)		PR^d^ n (%)	
	p-value <0.001		p-value <0.001			p-value <0.001		p-value <0.001	
	High	Low	Low	Intermediate	High	Low	High	Low	High
**Histology**									
CAH	0	55 (100)	23 (40.4)	28 (49.1)	6 (10.5)	0	57 (100)	1 (1.8)	55 (98.2)
EEC Grade 1	11 (6.4)	162 (93.6)	19 (12.3)	95 (61.7)	40 (26.0)	18 (9.3)	175 (90.7)	12 (6.2)	182 (93.8)
EEC Grade 2	23 (15.8)	123 (84.2)	29 (23.0)	63 (50.0)	34 (27.0)	34 (21.7)	123 (78.3)	20 (12.7)	137 (87.3)
EEC Grade 3	11 (20.4)	43 (79.6)	9 (19.6)	20 (43.4)	17 (37.0)	21 (36.8)	36 (63.2)	22 (38.6)	35 (61.4)

a Index 9 indicated as high, 0-6 is low, Cell Signaling (#3352).

b Low is index 0, intermediate is 1-4, high is 6-9, Cell Signaling (#9188).

c Index 0-2 is low, 3-9 is high, Dako (M7047).

d Index 0 is low, 1-9 is high, Dako (M3569). Missing scoring data in two cases with CAH for Stathmin and one case for PR. Missing scoring data for ER in one, PTEN in 82 and Stathmin in 35 cases with EEC. Statistic assessed by Chi-square test.

### Hormone receptor loss and epithelial to mesenchymal transition are rare in CAH

We further explored the primary investigation series for changes in mRNA expression levels for the genes encoding the hormone receptors ER and PR (*ESR1* and *PGR*), as well as an Epithelial-Mesenchymal Transition (EMT) signature score, hypothesizing that this transition could be important for the early invasive step in carcinogenesis. We found however, that the most significant reduction in hormone receptor level and increase in EMT score were detected from grade 2 to grade 3 EEC, with no significant change from CAH to grade 1 EEC (Supplementary Fig. S1). The observation that the proportion of cases with low hormone receptor level increases from low to high grade EEC lesions, and that the increase is clearly most pronounced during dedifferentiation after the invasive step is validated in the larger cohort of 57 CAHs compared to 408 EEC (Table 1). We found ERα and PR protein expression to be present in the majority of premalignant lesions, with loss of expression in less than 2% (Table 1).

### Oncogenic pathway alterations differ between obese and non-obese CAH patients

**Figure 4:**
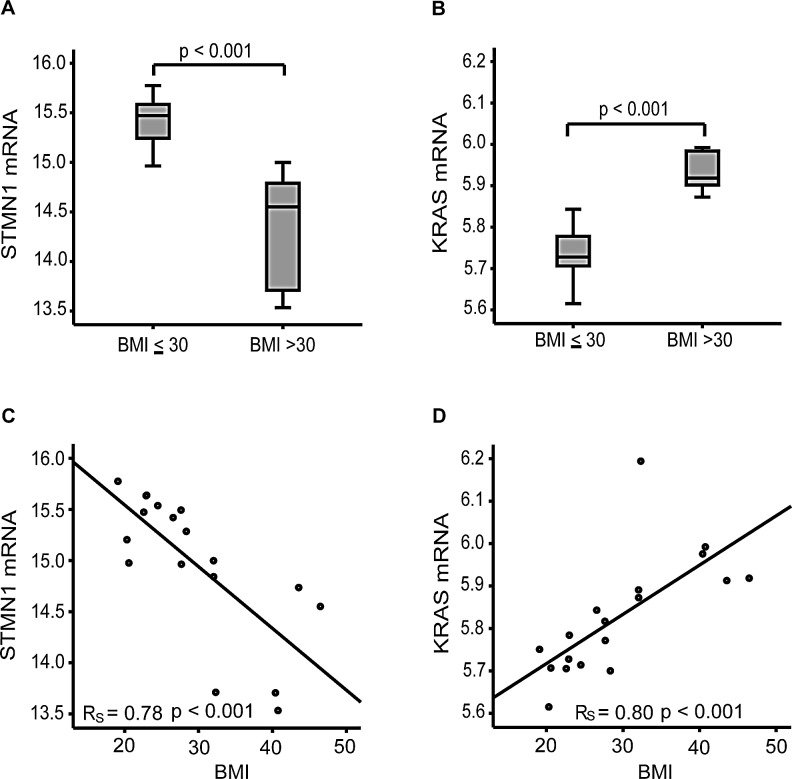
mRNA expression levels of *STMN1* and *KRAS* in relation to body mass index in CAH patients *STMN1* expression is significantly anticorrelated (A) and *KRAS* significantly correlated (B) with BMI in CAH patients. Distribution of *STMN1* and *KRAS* mRNA expression in 18 cases of complex atypical hyperplasia (CAH) related to body mass index (BMI) (C, D).

Obesity is a known risk factor for CAH and EEC [2]. Hypothesizing that the molecular alterations critical for cancer initiation are obesity-related, we explored gene expression in obese compared to non-obese patients in fresh frozen samples from CAH patients. Interestingly, we found *KRAS* and *STMN1* mRNA levels to be amongst the top-ranked differentially expressed genes in a SAM analysis, comparing women with BMI above and below 30 kg/m^2^. We found that mRNA expression of *STMN1* is significantly anti-correlated with BMI, contrasting *KRAS* mRNA expression being highly significantly correlated with high BMI (Figure 4). This strong association with BMI observed in the CAH group was not present in invasive EEC, suggesting that the molecular changes important in the initiation of the carcinogenic process in premalignant lesions are BMI context related (Supplementary Fig. S2). Although of borderline significance, we also observed the same tendency as for *STMN1* with higher PI3K activation score in non-obese compared to obese EEC patients (p=0.058).

We further explored the relationship between BMI and transcriptional alterations by applying Gene Set Enrichment Analysis (GSEA), using pre-defined gene sets from the MsigDB (http://www.broadinstitute.org/gsea/index.jsp) to compare obese and non-obese CAH patients. In line with the initial observation that *KRAS* level was positively correlated with BMI, obese patients with CAH demonstrate a striking enrichment for oncogenic *KRAS* related gene sets (Supplementary Table S4), this was not observed in EEC. In addition, an oncogenic *KRAS* related gene set generated in breast cancer, another hormone and obesity related cancer [5], clearly demonstrated a different activation pattern for non-obese compared to obese CAH patients (Supplementary Fig. S3). Also, gene sets related to immunological processes in Gene Ontology (GO) gene sets, were enriched in obese compared to non-obese CAH patients (Supplementary Table S4), a pattern that was not seen for invasive EEC. Due to the small sample size for the premalignant subgroup, we re-explored the data and doubled the permutations, without observing any change in number of significant gene sets, supporting the validity of our primary results.

### Targets for treatment differs between obese and non-obese patients

Further hypothesizing that optimal drugs for preventing and treating premalignant and low grade endometrial cancers may be different for obese and non-obese women, we queried Connectivity map (version 2) [21] for compounds anticorrelated to EEC grade 1 compared to CAH, also stratified for BMI groups. PI3K/mTOR inhibitors were found to be amongst the top ranked drugs, and thus suggested as treatment for patients with early EEC, and for the non-obese group in particular (Supplementary Table S5). This appears to be in line with the initial observation that *STMN1* and PI3K pathway activation increase from CAH to EEC, in particular amongst non-obese patients (Supplementary Table S5). This is also supported by higher Stathmin protein expression in the non-obese compared to the obese EEC patients in the validation cohort (p=0.03). We also found pStathmin(S38) to be significantly higher in the non-obese patients (p=0.007).

### Stathmin overexpression was frequently detected in preoperatively undetected EEC

Biomarkers that could identify cases with occult carcinoma, having a preoperative biopsy diagnosed as CAH, would be clinically useful. We therefore compared preoperative samples from 44 patients with EEC in hysterectomy specimens diagnosed as CAH in preoperative biopsies (discordant cases) to the 72 patients with CAH in both specimens (concordant cases). We find that Stathmin overexpression in preoperative biopsies is significantly more frequent in the discordant group (p=0.03), suggesting a potential to improve the diagnostic accuracy for detecting malignant disease preoperatively (Table 2). In line with these findings, there was a tendency, although not statistically significant, to higher pStathmin(S38) in discordant cases (p=0.06). Low PR protein expression also tended to be more common in preoperative biopsies for discordant compared to concordant cases (p=0.05). ERα and PTEN expression however, were similar for the two groups (Table 2).

**Table 2:** Protein expression in preoperative samples: Hormone receptors, Stathmin and PTEN in 44 patients classified as complex atypical hyperplasia (CAH) in preoperative biopsies with endometrioid endometrial carcinoma (EEC)* after hysterectomy (CAH/EEC); and 72 patients with concordant pre and postoperative diagnosis of complex atypical hyperplasia (CAH/CAH)

	Stathmin^a^ n(%)		PTEN^b^ n(%)			ERα^c^ n(%)		PR^d^ n(%)	
	p-value=0.03		p-value=0.37			p-value=0.55		p-value=0.05	
	High	Low	Low	Intermediate	High	Low	High	Low	High
**Histology**									
CAH/CAH	2 (2.9)	66(97.1)	41 (59.4)	27(39.1)	1(1.4)	1 (1.4)	68(98.6)	1 (1.4)	71(98.6)
CAH/EEC	7 (15.9)	37(84.1)	30 (71.4)	11(26.2)	1(2.4)	2 (5.0)	38(95.0)	4 (10.0)	36(90.0)

a Index 6-9 is regarded as high, 0-4 as low, Cell Signaling (#3352), missing data for four cases of CAH.

b Low is index 0, intermediate is 1-4, high is 6-9, Cell Signaling (#9188), missing data for two case of EEC and three cases of CAH.

c Index 0-2 is low, 3-9 is high, Dako (M7047), missing data for four cases of EEC and three cases of CAH.

d Index 0 is low, 1-9 is high, Dako (M3569), missing data for four cases of EEC. Statistical calculation: Fisher exact test was used, except for PTEN were Chi-square test was applied.

* One case with carcinoma displayed a carcinosarcoma in final hysterectomy specimen.

## DISCUSSION

The search for diagnostic markers and conservative treatment opportunities for patients with premalignant endometrial lesions and low grade malignant endometrial cancer is of importance as the incidence of these lesions are increasing. The need for non-invasive treatment opportunities is especially warranted in younger women wanting to preserve fertility. Currently hysterectomy is the preferred treatment for patients with atypical hyperplasia, due to risk of occult carcinoma, as well as the substantial risk of progression to invasive carcinoma [9, 22, 23]. In our material CAH patients were significantly younger than EEC patients. This appears to be in line with the knowledge of CAH as a condition preceding EEC, also supported by a nested case-control study demonstrating a cancer progression interval to be four to six years after initial diagnosis of complex or atypical hyperplasia [24].

Obesity is an increasing concern in both developed and less developed countries, and the link between high BMI, diet and cancer is a growing field of interest [5, 25]. Endometrial cancer, and especially endometrioid subtype and its precursor lesions, is strongly linked to obesity [5]. Interestingly we find that *KRAS* mRNA expression is significantly higher in obese patients with CAH, opposed to *STMN1* expression, being significantly higher in non-obese CAH patients. Previously, *KRAS* alteration has been reported to be an early event in the endometrial carcinogenesis [26], and *KRAS* mutations have been associated with higher BMI [27], apparently in line with our finding that *KRAS* seems to play a different role in obese compared to non-obese patients in the earliest premalignant part of the carcinogenesis. We have demonstrated here a distinct enrichment of *KRAS* related oncogenic signatures and GO signatures linked to inflammation in obese compared to non-obese CAH patients. Apparently in line with our findings, inflammation in general, and particular obesity induced inflammation processes are believed to be important in cancer initiation, promotion and metastatic progression [28]. Furthermore, obesity related immunological processes have been linked to *KRAS* induced cancer initiation of pancreatic adenocarcinoma in mice [29, 30]. Intriguingly, metformin, an anti-diabetic drug used to treat metabolic syndrome and obesity related diabetes, has been shown to inhibit cell proliferation in endometrial cancer cells with activated *KRAS,* further suggesting a link between *KRAS* status and the potential for targeting metabolic syndrome in obese patients with endometrial carcinoma (precursor) lesions [31]. Also, based on studies of obese rats, metformin has been demonstrated to decrease estrogen-related endometrial proliferation, partly through the MAPK/ERK pathway [32]. In our small set of 10 patients assessed by WES we found no *KRAS* mutation. Whether the observed *KRAS* activation in obese patients with CAH lesions could be explained by increased upstream signaling, post-translational modification or loss of DNA methylation needs further studies. We found that *STMN1* and PI3K pathway activation increase before invasion in non-obese patients in particular, indicating their key-role in the initiation of carcinogenesis in the non-obese context. This is also in line with the clinical observation that tumors less related to hormonal risk factors more often demonstrate activated PI3K signaling [16] and are more common in non-obese women.

We demonstrate here a late loss of hormone receptors, both on mRNA and protein level in the endometrial carcinogenesis. In addition, a significant increase in a predefined EMT gene signature [33] seem to occur during dedifferentiation. This is in line with earlier findings proposing EMT as a late event in endometrial carcinogenesis, also linked to loss of hormone receptors [33]. Hormone receptor expression has, also in line with our results, previously convincingly been linked to the endometrioid subtype and is more common in low grade compared with high grade lesions [34]. Loss of PR protein expression has recently been demonstrated to be an independent negative prognostic marker in patients with endometrioid endometrial cancer. Loss of protein expression increase from premalignant to malignant lesions and further during dedifferentiation [35]. Premalignant endometrial lesions, as well as low grade endometrioid malignant lesions often demonstrate preserved hormone receptor expression. Also, high expression of PR is proposed as a marker for progestin therapy response in patients with premalignant endometrial lesions [36]. The epigenetic mechanisms behind PR silencing have been suggested to differ between well differentiated and poorly differentiated endometrial cancer cell lines. Also, in early stage and grade endometrial cancer lesions miRNA seems important in post-transcriptional repression of PR [37]. Recently *HAND2* methylation has emerged as a key step in the early endometrial carcinogenesis [38]. Aberrant DNA methylation is already detected in normal endometrial samples from women with CAH, and has been suggested as a biomarker of early detection of endometrial cancer. *HAND2* is influenced by progesterone, also supported by the finding that *HAND2* methylation status predicts response to progestin therapy [38]. Still, the clinical value of *HAND2* methylation assessment needs to be further investigated. Another recent and interesting observation is that the DNA methylation status of the testis specific gene CTCFL/BORIS is linked to the level of PR expression in endometrial cancer and premalignant lesions, however causality needs to be explored [39].

The preoperative distinction between premalignant and low grade malignant endometrial lesions is challenging, and the reproducibility of the diagnosis have been found to be low, even among expert pathologist [7, 8]. The preoperative diagnostic criteria applied, as well as patient age and lesion characteristic, influence the proportion of undetected carcinoma [40]. Even so, further search for additional biomarkers for clinical use is required. We find Stathmin protein expression to be significantly higher in endometrial carcinomas preoperatively diagnosed as CAH. Also high pStathmin(S38) and low PR protein expression tended to be more common in discordant cases. Others have reported significantly lower expression of both ERα and PR in patients with concurrent carcinoma [41]. Further, as we demonstrate here, others have also reported assessment of PTEN expression as inadequate in predicting concurrent carcinoma [42]. Likewise, other immunohistochemical patterns including hormone receptors, COX-2, Mlh1 and Bcl-2 have been explored in the clinical setting as potential biomarkers, but without added value to the traditional histology assessment [41, 43]. For premalignant endometrial lesions the morphometric classification system of *Endometrial intraepithelial neoplasia* (EIN) has been suggested as a more objective and reproducible diagnostic tool, and superior to the WHO-system in predicting concurrent carcinoma or cancer progression [44-46]. This have led to a revised binary modification of the WHO classification recently published, hopefully to improve diagnostic reproducibility [11].

To our knowledge this is the first time CAH lesions have been characterized by WES. Although the number of included patients is small, *PIK3CA* and *PTEN* were significantly mutated, in line with earlier reports [47, 48]. Mutations in *KRAS* and *CTNNB1*, previously reported to be present in premalignant lesions and thus suggested as early events in the endometrial carcinogenesis [49, 50], were not detected in our material. This may be due to the relatively small sample set investigated in the current study by WES. Recently *CTNNB1* mutation within exon 3 and related Wnt/β-catenin pathway activation have been linked to a subgroup of low grade and low stage EEC exhibiting a worse outcome [51]. *Ctnnb1* has been demonstrated to be important in normal endometrial epithelial function, and dysregulation has been linked to squamous cell metaplasia in mice [52]. Also, knowing that *CTNNB1* is regarded a premalignant phenomenon in endometrial carcinogenesis makes this pathway highly relevant to scrutinize in the premalignant setting in a larger cohort.

We validated and expanded the number of cases investigated for *PIK3CA* mutations by applying Sanger sequencing of the hot spot regions exons 9 and 20. Six *PIK3CA* mutations in altogether 20 cases with CAH were detected (including one silent mutation in exon 20), of which 10 cases were characterized by WES. In endometrial carcinogenesis *PIK3CA* mutations have previously been linked to invasion [47], while high frequency of *PIK3CA* amplification has been linked to dedifferentiation and aggressive phenotype [16] in line with our present findings. Mutation and amplifications are also considered to be independent events, where both may lead to PI3K pathway activation [15]. In breast carcinoma, apparently in line with our findings in endometrial carcinoma (precursor) lesions, mutation in *PIK3CA* is assumed to be an early event and associated to favorable prognosis [53, 54]. However, *PIK3CA* mutations are, by some, regarded as a late event in endometrial carcinogenesis, rarely found in premalignant lesions [47, 48]. In endometrial cancer, the reported effects of *PIK3CA* mutations are inconsistent, although mostly reported to be unrelated to outcome, also supported by being more frequent in grade 1 EEC compared to higher grade [16, 17, 47], others have reported specific *PIK3CA* mutations in exon 20 to associate with adverse prognostic parameters [55]. Our finding of 30% (6/20) of the examined premalignant lesions found to harbor *PIK3CA* mutations was surprising. Knowing that the distinction between premalignant and malignant lesions in the endometrium is challenging, introduces an uncertainty related to the conclusion. Also for our data, the report generated by one pathologist may be subjective, and such distinction between premalignant and malignant lesions may be inaccurate, although representing the routine clinical practice. Also our assessment of *PIK3CA* mutations targeted by Sanger sequencing is limited by the fact that only exon 9 and 20 are included. In endometrial cancer, several hot spot mutations are found within these two exons, but they still represent only around half of the *PIK3CA* mutations reported [56].

*PTEN* mutations are amongst the most commonly reported genetic alterations in the EEC, and have been reported to associate with favorable survival [57], although the effect on outcomes remains controversial. Apparently in line with PTEN being a marker for more favorable outcome, loss of PTEN protein expression has been demonstrated to implicate good prognosis in endometrial cancer, especially in obese patients [58, 59]. However conflicting results for protein assessment of PTEN status by IHC in prognostication has diminished its clinical utility [60]. In mice, knockout of PTEN has been demonstrated to be an important early event in the initiation of the endometrial carcinogenesis, possibly also relevant in humans [61]. *PTEN* mutations have also been reported to be present in a substantial portion of premalignant lesions of the endometrium, however one particular *PTEN* deficient cell clone is usually not persistent, but frequently replaced by other *PTEN* deficient clones over time [62, 63]. In our material, loss of PTEN protein expression is more frequent in premalignant lesions, consistent with PTEN loss being an early event, also known to occur in normal endometrium [62, 63]. Our finding that 40% of CAH lesions display PTEN loss is in line with previous reports [64].

PTEN aberrations, both mutations and loss of protein expression, have been demonstrated as preinvasive events [62, 64, 65]. *PIK3CA* mutations, on the other hand, have been reported to be infrequent or absent in premalignant endometrial lesions [47, 48]. We have here demonstrated molecular changes in *PIK3CA* and *PTEN* to be present already in the precursor lesion for EEC, with transcriptional changes in the PI3K pathway activation as early events in the invasive step to grade 1 EEC. These changes from precursor lesions to invasive cancers were confirmed by immunohistochemical staining for Stathmin, supporting the use of this protein as a surrogate marker for PI3K signaling [16, 19]. Mutations in *PTEN* and *PIK3CA* often coexist in EEC, and simultaneous mutations in these two genes have been linked to invasion [47]. Nevertheless, we find that two cases with CAH have this combination of mutations.

For assessing PTEN status, IHC has been found to be superior to sequencing in detecting loss of function [66], and in line with this, we find *PTEN* mRNA expression and protein level to be highly correlated. Thus, our results support that assessment of PI3K signaling and PTEN loss in FFPE tissue, by IHC staining for Stathmin and PTEN, respectively, is valid and more readily available for use in a routine clinical setting.

We conclude from the present study, to our knowledge the most comprehensive molecular characterization of specimens ranging from precursors through primary to metastatic EEC lesions, that *PTEN* mutations and loss, mutations in *PIK3CA* as well as PI3K and *KRAS* signaling activation are early events in the development from CAH to EEC, while hormone receptor loss and EMT occur during dedifferentiation. Interestingly, the molecular alterations in precursor lesions are BMI context related. *KRAS* and immunological activation is predominantly prevalent in obese women, while *STMN1* and PI3K signaling activation is prevalent in non-obese women, suggesting different prevention and systemic treatment strategies in obese and non-obese patients.

## METHODS

### Patient samples

Tumor specimens were collected from the Bergen Gynecologic Cancer Biobank, and linked to comprehensive clinical and histopathological data as previously reported [67]. Patients were diagnosed and treated by hysterectomy at Haukeland University Hospital between May 2001 and July 2013.

### Investigation cohort

Fresh frozen tissue from 18 CAH, 139 primary EEC and 17 metastatic lesions from EECs (one without corresponding primary tumor) were applied for comprehensive molecular profiling.

### Validation cohort

Under the assumption that CAH is precursor lesions for the endometrioid histologic subtype, only patients with EEC were included for comparison with the CAH. 494 patients with EEC and 77 patients with CAH were prospectively included. From these, FFPE tissue from hysterectomy was available from 408 patients with EEC and 57 patients with CAH for further immunohistochemical studies and included in the analyses.

Both cohorts had clinical information including age at primary treatment, BMI, menopausal status, parity, histology and treatment available. For the carcinomas follow-up data, FIGO stage, histologic subtype and grade were recorded.

### Discordant CAH/EEC samples

Samples from 44 patients diagnosed with endometrial carcinoma in hysterectomy specimens that were discordant with the diagnosis CAH in preoperative biopsies, were explored for potential biomarkers that could improve the preoperative detection of malignant disease, by comparing them to 72 cases with concordant CAH diagnosis in preoperative and hysterectomy samples.

This study was evaluated and approved according to Norwegian legislation and in line with international demands for ethical review. The study was approved by the Norwegian Data Inspectorate, Norwegian Social Sciences Data Services, and the Western Regional Committee for Medical and Health Research Ethics, (NSD15501; REK 052.01). Patients signed an informed consent.

Fresh frozen tissue was collected from patients included in the investigation cohort and used for DNA and RNA extraction as previously reported [27, 33].

### Immunohistochemical (IHC) staining

Tissue Micro Arrays (TMAs) were prepared and protein expression for ERα, PR, Stathmin and PTEN was assessed by immunohistochemistry as previously reported [34, 67-69] (Supplementary Method). The evaluation of the stained slides was conducted as described [34, 58, 67, 68].

### Fluorescent in Situ Hybridization (FISH)

*PIK3CA* gene copy number was assessed by FISH on TMAs using the Histology FISH Accessory Kit (DAKO) according to the manufactures recommendations with minor modifications (Supplementary Method).

### DNA sequencing

Fresh frozen tissue was used for DNA extraction. Corresponding normal DNA samples were obtained from blood or histological verified normal uterine tissue in cases where DNA from blood was unavailable. Whole exome sequencing (WES) was performed on ten cases with CAH and matching normal samples using an Illumina HiSeq 2000 platform with analysis following the pipelines at the institute of BROAD [12]. Sample coverage of targeted base pairs was at averaged 85% with a mean read length of 76x. Mutational significance for this group of samples was assessed by MutSig analysis http://www.broadinstitute.org/cancer/cga/mutsig. Sanger sequencing was applied for mutational analyses of *PIK3CA* for exons 9 and 20 in 18 cases with CAH (eight overlapping with the samples used for whole exome sequencing) and 228 cases of EEC. Details for the procedure and primers used are previously described [27, 33].

### Oligonucleotide DNA microarray analyses

Extracted RNA was hybridized to Agilent Whole Human Genome Microarrays (G4112F) according to manufacturer's instruction (http://www.agilent.com) as previously reported [33]. For single gene detection significance analysis of microarrays (SAM) was applied with default permutation set to 400 [70]. Gene set enrichment analysis was used to find relevant biological pathways [71], using a default permutation set to 1000. PI3K signature score was calculated using the gene expression signature reported by Gustafson *et al.* [18]. An EMT score was calculated as previously reported from EMT related genes [33]. For all gene signatures generated the expression values were mean normalized and scaled to the same SD [16]. Microarray data are publicly available at ArrayExpress, with accession number E-MTAB-2532.

### Reverse Phase Protein Array (RPPA)

Protein level of Stathmin and PTEN was assessed by RPPA in 9 patients with CAH and 242 EEC patients. The procedure was done as previously reported [72] and described briefly in Supplementary Methods.

### Statistical analyzes

The data was analyzed using SPSS (Statistical Package of Social Science) version 21.0. All p-values are two-sided and considered statistically significant when <0.05. The differences in gene expression values between groups were calculated using a non-parametric Mann-Whitney-U test. Categorical data were compared using Pearson X^2^ or Fisher exact test as appropriate. Correlations between continuous variables are indicated by Spearman correlation coefficient.

## SUPPLEMENTARY MATERIAL, FIGURES AND TABLES




