# Supplementary material for: Molecular profiling of endometrial carcinoma precursor, primary and metastatic lesions suggests different targets for treatment in obese compared to non-obese patients
Source: Oncotarget. 2014 Nov 4;6(2):1327–39. doi: 10.18632/oncotarget.2675 (PMC4359236; doi:10.18632/oncotarget.2675)
Supplement: Supplementary file 1 [file oncotarget-06-1327-s001.pdf]

Molecular profiling of endometrial carcinoma precursor, primary and metastatic lesions suggests different targets for treatment in obese compared to non-obese patients

Supplementary Material

A: Conditions applied for immunohistochemical staining.

Primary antibody	Clonality and origin	Vendor	Buffer	Incubation time	Incubation temperatur	Dilution
ERα	Monoclonal mouse	Dako (M7047)	pH 9	60 min	Room temp.	1:50
PR	Monoclonal mouse	Dako (M3569)	pH 9	60 min	Room temp.	1:150
PTEN	Monoclonal rabbit	Cell Signaling (#9188)	pH 6	Over night	4 °C	1:100
Stathmin	Polyclonal rabbit	Cell Signaling (#3352)	pH 6	30 min	Room temp.	1:50
pStathmin(S38)	Monoclonal rabbit	Cell Signaling (#4191)	pH 6	30 min	Room temp.	1:200

Slides were dewaxed and rehydrated. Next, the slides were subjected to microwave antigen retrieval and peroxidase treatment before incubation with the primary antibody, followed by secondary antibody before covered with DAB+ and counterstained by Haematoxylin.

B: Fluorescent in Situ Hybridization (FISH) performed as recommended by the manufacture, with minor modification as follows.

Slides were heated overnight at 58°C before deparaffinization 3×10 minutes in xylene. De- and rehydration was performed in three steps with 100%, 85% and 70% ethanol. Pepsin digestion was performed for 15 minutes. TMAs were incubated with Abnova PIK3CA/CEP3 Dual Colour probe at 75°C for 5 minutes and 37°C for approximately 72 hours. After screening all spots (3-9 spots per patient) from one case for copy number increase, taking the entire tissue of each spot into account, the area with optimal signal quality and quantity was selected for assessment of gene and centromere signals in 20 non-overlapping nuclei. Absolute mean PIK3CA gene copy number and average PIK3CA/CEP3 ratio was determined.

C: Reverse Phase Protein Array (RPPA) method in brief.

In brief, fresh frozen patient samples were homogenized in RPPA lysis buffer and protein lysate concentrations adjusted. Protein was denatured using SDS, followed by serial dilution in lysis buffer. Samples were printed on nitrocellulose-coated slides by an Aushon Biosystems 2470 arrayer. Slides were analyzed with primary antibodies thoroughly validated for RPPA, before corresponding secondary antibodies were applied using a BioGenix autostainer. Dako Cytomation-catalyzed systems and DAB colorimetric reaction were used for signal capturing. Spot signal intensities were quantitated using Arraypro software, following scanning of slides by CanoScan 9000F. Relative protein levels are determined by fitting each dilution curve with a logistic model ("Supercurve Fitting"), http://bioinformatics.mdanderson.org/OOMPA. Raw data was normalized before analysis.

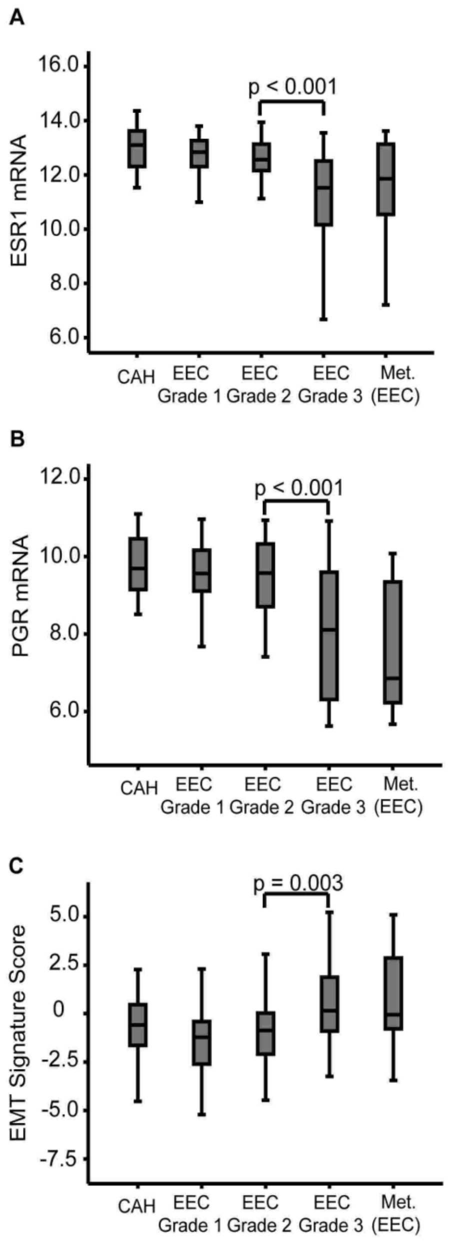

Figure S1: ESR1 and PGR mRNA expression and EMT signature score in CAH and EEC according to subtype and grade

Gene expression of ESR1 (A), PGR (B) and EMT signature seore (C) are displayed according to the five types of lesions studied: complex atypical hyperplasia (CAH), primary endometrioid endometrial carcinoma (EEC) stratified grade 1, grade 2 and grade 3 and metastatic lesions from EEC primary tumors.

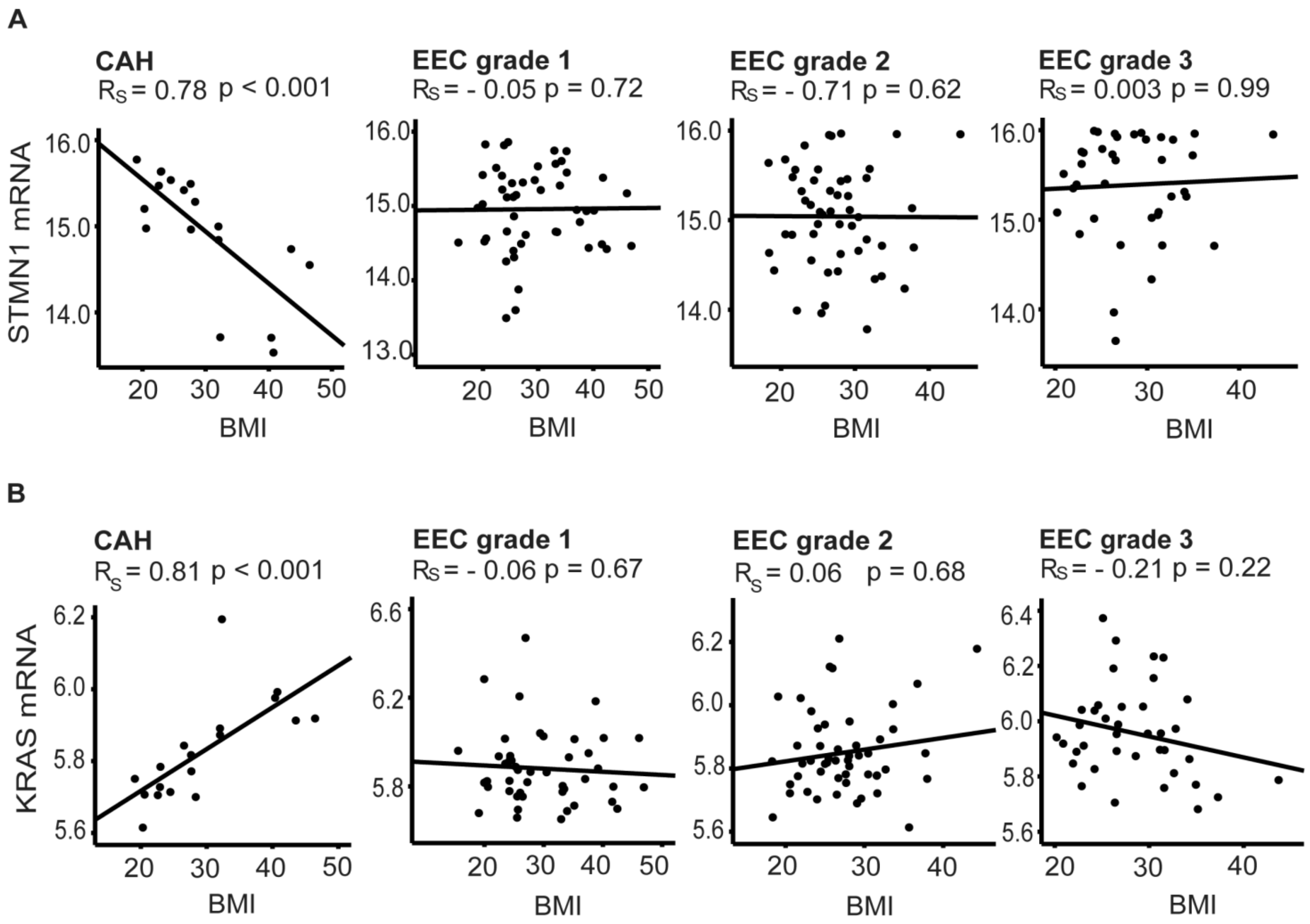

Figure S2: **Scatterplots presenting STMN1 and KRAS mRNA levels correlated to body mass index**

Distribution of STMN1 (A) and KRAS (B) mRNA levels for lesions with complex atypical hyperplasia (CAH) and endometrioid endometrial cancer (EEC) grade 1, 2 and 3, related to body mass index (BMI). Spearman correlation coefficient is reported for the correlation between continuous variables.

A

B

C

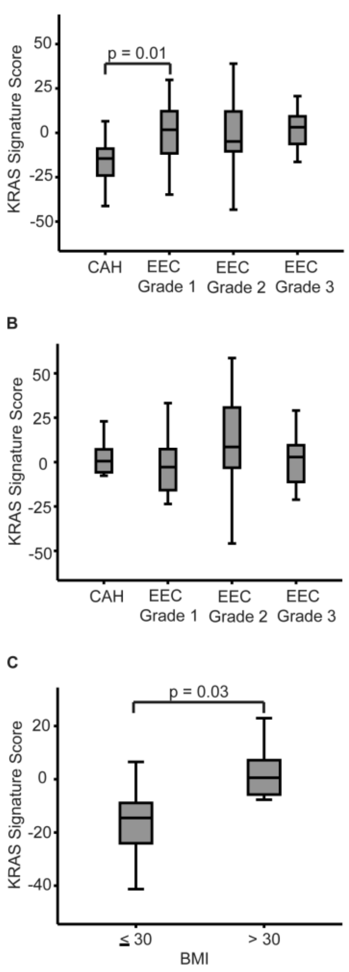

Figure S3: KRAS oncogene signature is increased in obese compared to non-obese patients with complex atypical hyperplasia (CAH)

KRAS related gene signature (KRAS.BREAST_UP.V1_UP vs KRAS.BREAST_UP.V1_DN) explored in complex atypical hyperplasia (CAH) and endometrioid endometrial cancer (EEC) patients stratified for grade 1, grade 2 and grade 3 in non-obese (BMI ≤30 kg/m^2) (A) and obese (BMI > 30 kg/m^2 (B) patients. The gene signature increase from CAH to EEC grade 1 lesions only for the non-obese group. KRAS related gene signature in CAH patients according to BMI (C).

Table S1: Distribution of clinical characteristics in patient cohorts. Investigation cohort: 139 patients with endometrioid endometrial carcinoma (EEC) and 18 patients with complex atypical hyperplasia (CAH). Validation cohort: 494 patients with EEC and 77 patients with CAH.

	Investigation Cohort				Validation Cohort			
**Variable**	**CAH/EEC Mean**	**CAH n (%)**	**EEC n (%)**	**p-value**	**CAH/EEC Mean**	**CAH n (%)**	**EEC n (%)**	**p-value**
**Age**[a]	62.4/65.1			NS[d]	61.2/63.9			0.02[d]
**BMI (kg/ m²)**[b]	29.5/28.4			NS[d]	29.5/28.9			NS[d]
**Para**[c]								
0		2 (11.1)	24 (17.3)	NS[e]		9 (11.8)	81 (16.5)	NS[f]
≥ 1		16 (88.9)	115 (82.7)			67 (88.3)	410 (83.5)	
**Menopausal status**								
Pre-		3 (16.7)	8 (5.8)	NS[e]		13 (16.9)	32 (6.5)	0.002[f]
Peri/post		15 (83.3)	131 (94.2)			64 (83.1)	462 (93.5)	

[a] Mean age at primary treatment. [b] Missing data for Body Mass Index (BMI) for CAH in 0/5 cases and in 1/49 with EEC in investigation samples/ validation samples. [c] Missing data for one patient with CAH and three with EEC in validation samples. [d] Mann Whitney U test. [e] Fisher`s exact test. [f] Chi-square test. NS (not significant).

Table S2: PIK3CA mutations in a total of 20 patients with CAH, assessed by whole exome sequencing (WES) and/or Sanger sequencing

Case	WES	Sanger sequencing
A	No mutation detected	No mutation detected
B	p.E542K	p.E542K
C	No mutation detected	No mutation detected
D	No mutation detected	No data
E	No mutation detected	No mutation detected
F	p.M1043V	No mutation detected
G	No mutation detected	No mutation detected
H	No mutation detected	No mutation detected
I	p.E545K	p.E545K
J	No mutation detected	No data
K	No data	No mutation detected
L	No data	p.Q546R
M	No data	No mutation detected
N	No data	No mutation detected
O	No data	No mutation detected
P	No data	p. E542G
Q	No data	p. T1025T(Silent)
R	No data	No mutation detected
S	No data	No mutation detected
T	No data	No mutation detected

Table S3: *PIK3CA* mutations analyzed in 18 cases with CAH and 228 cases with EEC. Five EEC cases have no information regarding grade. *PIK3CA* copy number alteration assessed by Fluorecent In Situ Hybridization (FISH) in 55 cases of CAH and 435 cases of EEC.

	***PIK3CA* mutation**[a]		***PIK3CA* amplification**[b]	
	n (%)	p-value*	n (%)	p-value**
**Histology**				
CAH	4 (22.2)	NS	0	0.01
EEC grade 1	12 (15.4)		8 (4.2)	
EEC grade 2	13 (13.7)		10 (6.0)	
EEC grade 3	7 (14.0)		20 (26.3)	

[a] Assessed by Sanger sequencing of PIK3CA gene, exon 9 or 20. [b] Assessed by FISH. * Fisher`s exact test used, ** Chi-square test.

Table S4: Top ranked gene sets enriched in obese patients with complex atypical hyperplasia (CAH) using Gene Set Enrichment Analysis (GSEA) from MsigDB. KRAS-related oncogenic gene sets are indicated by Gray color.

Rank	Oncogenic gene sets	FDR (%)	Gene Ontology (GO) gene sets	FDR (%)
1	RPS14_DN.V1_UP	0.0	IMMUNE_RESPONSE	0.0
2	P53_DN.V2_UP	0.1	IMMUNE_SYSTEM_PROCESS	0.0
3	HINATA_NFKB_IMMU_INF	0.1	G_PROTEIN_COUPLED_RECEPTOR_BINDING	0.0
4	IL2_UP.V1_UP	0.1	HUMORAL_IMMUNE_RESPONSE	0.0
5	ALK_DN.V1_UP	0.1	DEFENSE_RESPONSE	0.0
6	KRAS.600.LUNG.BREAST_UP.V1_UP	0.1	LOCOMOTORY_BEHAVIOR	0.0
7	KRAS.LUNG.BREAST_UP.V1_UP	0.2	CHEMOKINE_RECEPTOR_BINDING	0.0
8	RELA_DN.V1_DN	0.2	CHEMOKINE_ACTIVITY	0.0
9	HOXA9_DN.V1_UP	0.2	LEUKOCYTE_ACTIVATION	0.0
10	SINGH_KRAS_DEPENDENCY_SIGNATURE_	0.3	CELL_ACTIVATION	0.0
11	IL15_UP.V1_UP	0.4	LYMPHOCYTE_ACTIVATION	0.0
12	KRAS.LUNG_UP.V1_UP	0.5	T_CELL_ACTIVATION	0.0
13	KRAS.AMP.LUNG_UP.V1_DN	0.6	INFLAMMATORY_RESPONSE	0.0
14	PTEN_DN.V1_DN	0.6	CYTOKINE_METABOLIC_PROCESS	0.0
15	KRAS.LUNG_UP.V1_DN	0.6	BEHAVIOR	0.0
16	KRAS.BREAST_UP.V1_UP	0.9	CELLULAR_DEFENSE_RESPONSE	0.0
17	JNK_DN.V1_UP	0.9	CYTOKINE_BIOSYNTHETIC_PROCESS	0.0
18	KRAS.AMP.LUNG_UP.V1_UP	1.1	POSITIVE_REGULATION_OF_CYTOKINE_BIOSYNTHETIC_PROCESS	0.0
19	KRAS.600_UP.V1_DN	1.1	REGULATION_OF_CYTOKINE_BIOSYNTHETIC_PROCESS	0.0
20	KRAS.KIDNEY_UP.V1_DN	1.0	REGULATION_OF_T_CELL_ACTIVATION	0.0
21	WNT_UP.V1_DN	1.2	RESPONSE_TO_EXTERNAL_STIMULUS	0.0
22	VEGF_A_UP.V1_UP	1.4	CYTOKINE_PRODUCTION	0.0
23	KRAS.600_UP.V1_UP	1.5	REGULATION_OF_IMMUNE_SYSTEM_PROCESS	0.0
24	PTEN_DN.V2_UP	1.5	T_CELL_PROLIFERATION	0.0
25	BCAT.100_UP.V1_DN	1.1	RESPONSE_TO_WOUNDING	0.0

Table S5: Top ranked therapeutic agents in Connectivity Map comparing patients with endometrioid endometrial cancer (EEC) grade 1 to complex atypical hyperplasia (CAH) (A), and the same patients stratified for body mass index (BMI) ≤ 30 kg/m^2 (B) and BMI > 30 kg/m^2 (C).

	Rank	Name of compound	Description	n	p-value
A	1	**LY-294002**	PI3K inhibitor	61	< 0.00001
	2	**Tanespimycin**	HSP90 inhibitor	62	< 0.00001
	3	**Trichostatin A**	HDAC inhibitor	182	< 0.00001
	4	**Sirolimus**	mTor inhibitor	44	0.0004
	5	**Phthalylsulfathiazole**	Sulfonamide; anti-bacterial	5	0.005
B	1	**LY-294002**	PI3K inhibitor	61	< 0.00001
	2	**Sirolimus**	mTor inhibitor	44	< 0.00001
	3	**Phthalylsulfathiazole**	Sulfonamide; anti-bacterial	5	0.0001
	4	**Ethotoin**	Anti-epileptica	6	0.0002
	5	**Emetine**	Anti-parasitic	4	0.0004
C	1	**Trifluoperazine**	Dopamin antagonist	16	< 0.00001
	2	**Trichostatin A**	HDAC inhibitor	182	< 0.00001
	3	**LY-294002**	PI3K inhibitor	61	0.00004
	4	**Tanespimycin**	HSP90 inhibitor	62	0.00004
	5	**Pyrvinium**	Anthelminitic	6	0.0001
